# Neuromorphic Photonics Based on Phase Change Materials

**DOI:** 10.3390/nano13111756

**Published:** 2023-05-29

**Authors:** Tiantian Li, Yijie Li, Yuteng Wang, Yuxin Liu, Yumeng Liu, Zhan Wang, Ruixia Miao, Dongdong Han, Zhanqiang Hui, Wei Li

**Affiliations:** 1 School of Electronic Engineering, Xi’an University of Posts and Telecommunications, Xi’an 710121, China; 2 College of Chemistry and Molecular Engineering, Peking University, Beijing 100871, China; 3 Los Alamos National Laboratory, Computer, Computational, and Statistical Sciences Division, Los Alamos, NM 87545, USA

**Keywords:** phase change materials, neuromorphic photonics, silicon photonics

## Abstract

Neuromorphic photonics devices based on phase change materials (PCMs) and silicon photonics technology have emerged as promising solutions for addressing the limitations of traditional spiking neural networks in terms of scalability, response delay, and energy consumption. In this review, we provide a comprehensive analysis of various PCMs used in neuromorphic devices, comparing their optical properties and discussing their applications. We explore materials such as GST (Ge_2_Sb_2_Te_5_), GeTe-Sb_2_Te_3_, GSST (Ge_2_Sb_2_Se_4_Te_1_), Sb_2_S_3_/Sb_2_Se_3_, Sc_0.2_Sb_2_Te_3_ (SST), and In_2_Se_3_, highlighting their advantages and challenges in terms of erasure power consumption, response rate, material lifetime, and on-chip insertion loss. By investigating the integration of different PCMs with silicon-based optoelectronics, this review aims to identify potential breakthroughs in computational performance and scalability of photonic spiking neural networks. Further research and development are essential to optimize these materials and overcome their limitations, paving the way for more efficient and high-performance photonic neuromorphic devices in artificial intelligence and high-performance computing applications.

## 1. Introduction

Artificial intelligence (AI) technologies, including facial recognition, machine learning, and autonomous driving, are transforming daily life. Implementing task-specific AI necessitates training neural networks with large datasets using computers, which currently faces limitations in throughput and efficiency due to existing computer architectures [1]. Drawing inspiration from brain structures, researchers have proposed next-generation intelligent computing systems that simulate synapses and neurons. These systems encode information as spatio-temporal pulse patterns from presynaptic neurons, with postsynaptic neurons performing pulse accumulation and generating new neuronal pulses after reaching the stimulation threshold. By integrating a multitude of neurons, these systems form nonlinear spiking neural networks, realizing information processing through spatio-temporally encoded neuron pulses. Intel’s TrueNorth chips have successfully integrated millions of neurons on a single chip, achieving an energy efficiency two orders of magnitude greater than conventional microelectronic chips for specific AI tasks at a level comparable to the human brain [2]. However, electrical interconnect bandwidth, pulse loss, and communication delay still constrain the scale of integrated neurons. Optical interconnects, offering large bandwidth, low loss, and low latency, can address these electrical interconnect challenges. Neuromorphic photonic systems have been reported to achieve processing speeds 6–8 orders of magnitude higher than their electronic counterparts [3]. Silicon photonics, an optoelectronic integration technology compatible with well-established microelectronics technology [4], combines the ultra-large-scale logic and ultra-precision manufacturing characteristics of CMOS technology with the ultra-high speed and ultra-low power consumption advantages of photonic technology, resolving the contradiction between technological evolution and cost. Over the past several years, on-chip neural networks based on silicon photonic technology have rapidly advanced. In 2017, Shen et al. demonstrated an on-chip neural network using a silicon-based Mach–Zehnder interferometer structure capable of recognizing four fundamental vowels [5]. In this concept, an external subsystem sets the matrix element values for vector matrix multiplication based on Mach–Zehnder interferometer structures. To change these values during optimization, signals must be output from the neural network to the control system. Tait et al. proposed on-chip variable weight synapses based on silicon electro-optical modulators in 2016 [6], and on-chip neurons based on silicon electro-optical modulators in conjunction with off-chip multi-wavelength lasers, wavelength division multiplexers/demultiplexers, and on-chip photodetectors in 2019 [7]. This structure adjusts link weight by using electrical signals to modulate the silicon microring and controls the silicon microring modulator to achieve neuron function through electrical signals from on-chip detector optoelectrical conversion.

**Figure 1:**
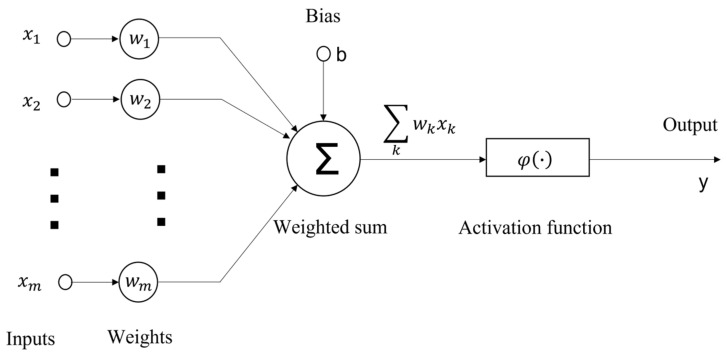
Nonlinear model of a neuron. The neuron is composed of a set of synapses (connecting links); an adder or linear combiner (performing a weighted sum of signals); and a nonlinear activation function [8].

The leaky integrate-and-fire (LIF) neurons are the most widely used spiking models in computational neuroscience. The LIF model incorporates properties derived from biological neurons, including a dendritic tree that collects, weights, and delays spiking signals from other neurons, a soma that temporally integrates these signals, and an axon that generates spikes when the integrated signal surpasses a threshold, as illustrated in Figure 1. The connections between neurons are referred to as synapses, while the strength of these connections is termed as weights. LIF neuron networks are Turing complete and, in principle, can perform any algorithm [8]. Silicon-based optoelectronic neuromorphic devices offer higher pulse time resolution, reduced communication delays and losses, and the potential for large-scale distributed neural network architectures [9,10,11]. Mature silicon waveguide devices, such as microring resonators, Mach–Zehnder interferometers, and wavelength division (de)multiplexers, are capable of simulating variable-weight synapses and spiking signal integration. In the LIF neuron model, the input-output nonlinear functional relationship between pre- and post-synapses is a critical mechanism for information processing and brain-like intelligence in neuronal models. However, most silicon photonic devices rely on linear optical effects. Implementing nonlinear mapping on silicon photonics chips requires nonlinear optical materials such as saturable absorption materials [12] or phase change materials (PCMs) [13]. Saturable absorbers exhibit a property of nonlinear dynamical mechanisms underlying all-or-none responses to small perturbations. An excitable LIF neuron can be physically realized using a vertical-cavity surface-emitting laser (VCSEL) combined with a saturable absorber (SA) in a cavity structure [14]. A photonic spiking neuron chip, based on an integrated Fabry–Perot laser with a saturable absorber (FP-SA), is capable of performing classification tasks using a supervised learning algorithm [15]. The integration of lasers and saturable absorption materials, such as graphene [14,16] and C_60_ [17], enables simulation of the inference process using externally imported weighted spikes. PCMs exhibit threshold switching and progressive crystalline/amorphous transition properties [18]. Compared to saturable absorption materials, PCMs are suitable for simulating both synaptic plasticity and integrate-and-fire functions of neurons, potentially leading to a more streamlined and scalable network structure.

**Figure 2:**
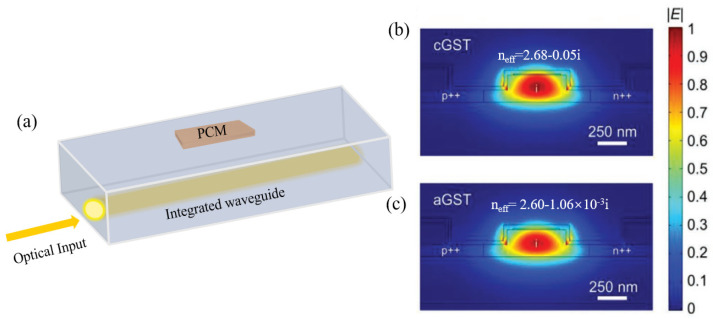
Neuromorphic photonic devices utilizing PCMs and integrated waveguides. (**a**) A 3D schematic representation of the hybrid waveguide. (**b**) Simulated electric field profiles for the fundamental quasi-TE mode of the switching unit at 1550 nm in the crystalline state (n_eff_ = 2.68 − 0.05i), and (**c**) the amorphous state (n_eff_ = 2.60 − 1.06 × 10^−3^ i) [19]. p++: p-type heavily doped region, n++: n-type heavily doped region, cGST: crystalline GST, aGST: amorphous GST.

Typically, chalcogenide-based phase-change materials are deposited onto integrated waveguides through sputtering, followed by a liftoff process. Rectangular openings can be defined on top of the waveguides by aligning them with previously written waveguide structures. A thin passivation layer, such as ITO or Al_2_O_3_, is also deposited to prevent oxidation. As shown in Figure 2a, the optical signal can then be input from the integrated waveguide and evanescently coupled into the phase-change units. When the optical power is high enough, a phase transition occurs, causing changes in the transmission amplitude and phase, accordingly, as depicted in Figure 2b,c [19].

Neuromorphic photonic devices based on PCMs and silicon photonics technology have the potential to overcome the scale limitation, response delay, and energy consumption issues associated with pulse transmission in traditional pulsed neural networks. Furthermore, these devices could help break the bottleneck of computing efficiency, memory, input/output, and energy consumption in electronic systems related to artificial intelligence and other high-performance computing fields. This work reviews the most promising neuromorphic devices based on PCMs, comparing their optical properties and discussing their various applications.

## 2. Neuromorphic Devices Development

**Figure 3:**
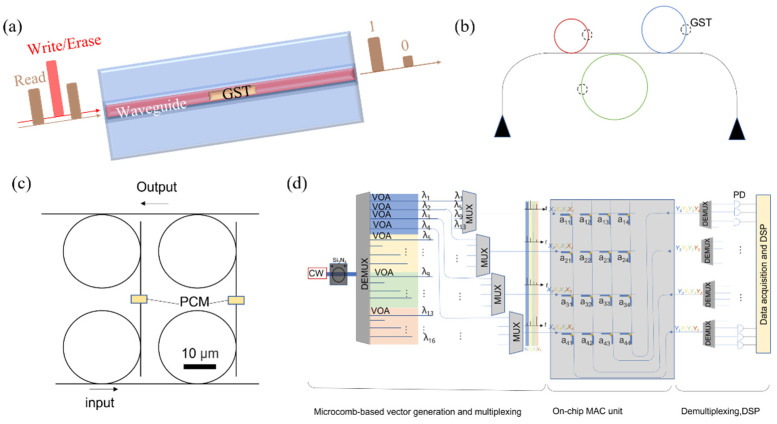
Photonic memory using optical phase change materials. (**a**) The schematic diagram of integrated on-chip memory. Information is stored in the phase state of the GST section atop the waveguide. Ultrashort optical pulses enable both reading and writing of the memory, as the guided light interacts with the GST through its evanescent field. During readout, data are encoded in the amount of optical transmission through the waveguide, as the two crystallographic states of GST display a high contrast in optical absorption [13]. (**b**) The multi-bit, multi-wavelength architecture realized by three different microrings coupled with the same waveguide. Using optical pulses close to resonance, each cell could be addressed selectively [13]. (**c**) A single memory cell within the 16 × 16 photonic matrix memory [22] (**d**) Schematic of multiplexed all-optical matrix-vector multiplication [23].

As a key component of on-chip optical neural networks, large-scale on-chip integrated optical storage has attracted significant research interest. In 2013, Ríos et al. demonstrated the first tunable photonic devices that integrated photonic circuits with phase change materials [20]. They deposited nanoscale germanium antimony telluride (Ge_2_Sb_2_Te_5_, GST) films, which were capped with a thin layer of indium tin oxide (ITO), onto silicon nitride racetrack microring resonators and Mach–Zehnder interferometers. Due to the refractive index difference between amorphous and crystalline GST, the optical parameters, such as the Q-factor, resonance wavelength, and extinction ratio, change according to the phase change materials’ state. Although non-volatile switching was only achieved through annealing, the hybrid platform demonstrated significant potential for non-volatile integrated photonic memory. In 2015, researchers realized up to eight levels of bit storage in a single device composed of a Si_3_N_4_ waveguide and a 10 nm thin GST film [13]. This construction is depicted in Figure 3a. To achieve in-line device ”write/erase” operations, they proposed a multiple optical pulse programming method using varying energy—a high-power short pulse followed immediately by a long series of pulses with linearly decreasing power. This strategy enabled GST crystalline-amorphous transition, eliminated local amorphization risk during crystallization, and allowed hundreds of repeated switches between the two states. By precisely controlling the crystallization fraction of GST using this method, the device exhibited eight storage states (3-bits). Moreover, the structural design of three differently-sized microrings coupled to the same waveguide, as illustrated in Figure 3b, enabled three wavelengths of ”write/erase” operations, paving the way for wavelength division multiplexing integrated devices for high-density optical storage. Fine-tuning the ”write/erase” optical pulses and increasing the GST footprint allowed researchers to achieve up to 34 levels of storage (over 5-bits) in 2018 [21].

Feldmann et al. [22] demonstrated a photonic memory in an array format consisting of 16 × 16 elements. They integrated 256 all-photonic phase-change memory cells on a 1000 × 2400 μm^2^ Si_3_N_4_ chip, which can store 512 bits of data. In Figure 3c, each memory cell is composed of input and output waveguides and two microring resonators coupled to them. The two microring resonators are connected through a straight waveguide, with the GST placed in the middle of the straight waveguide as the storage medium. When light passes through the input waveguide, some of it is coupled to the lower microring resonator and the PCM waveguide. Once the light passes through the PCM and senses a phase transition, it is guided to the second microring resonator, which has the same radius, and consequently, the same resonance wavelength. The operational wavelength directed to the PCM is selected by adjusting the radius of the microring resonators for different memory cells. This design enables wavelength multiplexing and demultiplexing of the same input signal. The amount of light entering the PCM and the output waveguide is controlled by adjusting the gap between the resonators and the waveguide. The chip implements image storage with 16 × 16 pixels, with all units individually addressable and capable of reproducing the stored pixel map with high accuracy.

Conventional optical computing methods are severely limited by the lack of integrated non-volatile photonic memory and multiplexing capabilities for such calculations. In-store photonic computing with wavelength division multiplexing capability can be achieved by designing computationally dedicated integrated photonic tensor cores consisting of phase-change memory arrays that combine the on-chip storage characteristics of the storage convolution cores with photonic chip-based frequency comb technology, as shown in Figure 3d [23]. This photonic core design demonstrates the potential for light-speed computing at very low power for a variety of applications, ranging from real-time video processing to autonomous driving and artificial intelligence assistance.

**Figure 4:**
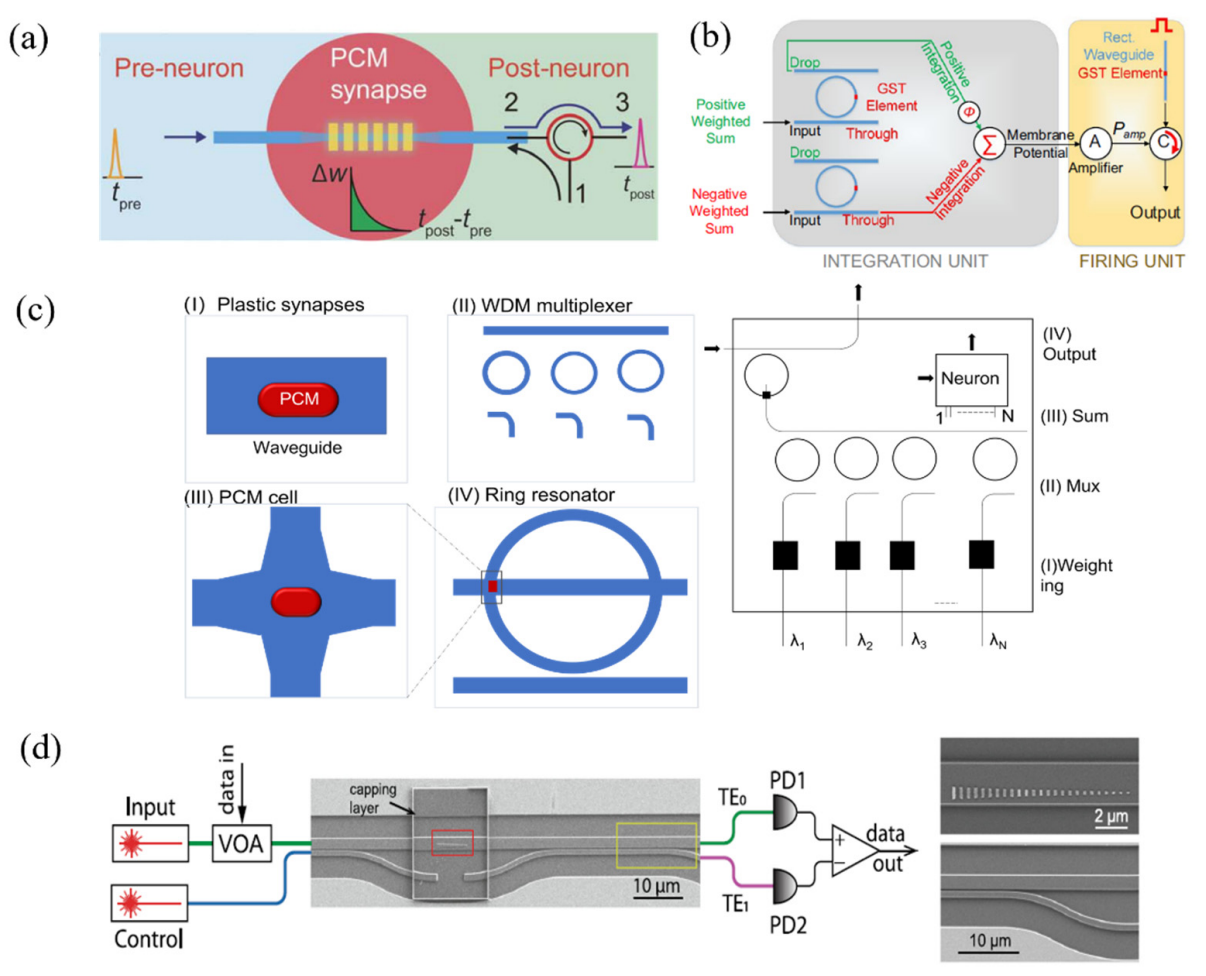
Neuromorphic devices based on GST. (**a**) Schematic of the integrated photonic synapse, which emulates the function of a neural synapse. The synapse comprises a tapered waveguide (dark blue) with discrete PCM islands on top, optically connecting the presynaptic (pre-neuron) and postsynaptic (post-neuron) signals [24]. (**b**) Schematic of a bipolar integrate-and-fire neuron based on GST-embedded ring resonator devices, illustrating the integration and firing unit [25]. (**c**) All-optical spiking neuronal circuits: Input spikes are weighted using PCM cells and summed up with a wavelength division multiplexer. When the integrated power of the postsynaptic spikes exceeds a certain threshold, the PCM cell on the ring resonator switches, generating an output pulse (neuronal spike) [26]. (**d**) The complete programmable metasurface mode converter device consists of an encapsulated GST phase gradient metasurface (red box) and a mode selector (yellow box) [27].

Neuromorphic devices based on nanophotonic technology and phase-change materials have also received considerable attention. In 2017, Chen et al. examined integrated photonic synapses by employing segmental deposition of GST in integrated silicon nitride waveguides [24], as illustrated in Figure 4a. The crystalline state of GST is used as the original state, where the device transmittance is defined as the baseline of the reading and assigned a synaptic weight of “0”. The change in synaptic weight corresponds to the change in transmittance of the reading relative to the baseline during the measurement, and the synaptic weight can be altered by sending an optical pulse along the waveguide. By using the pulse width modulation method for the PCM, a set of known pulses can ensure any arbitrary synaptic weight level without attention to the actual current weight. To mimic spike timing-dependent plasticity (STDP) behavior, the presynaptic signal is split into two beams, with 50% coupled into the photonic synapse and the other 50% (P_in1_) connected to the interferometer via a phase modulator. The postsynaptic signal is also divided into two parts, where 50% of the signal continues to be transmitted, and the rest (P_in2_) is fed back to the interferometer. Changing the phase modulator adjusts the net output power of the interferometer between 0 and P_in1_ + P_in2_, which is used to update the synaptic weights. With no time delay between pre- and postsynaptic spikes, the net output power from the interferometer applied to the synapse has a single pulse larger than the phase change threshold power. Increasing the time delay between the pre- and postsynaptic spikes alters the net output power, and thus, a different number of pulses are sent to the synapse. This results in the desired exponential dependence of synaptic weight changes on time delay, allowing for the efficient emulation of spike time-dependent plasticity behavior in a simple manner.

Another approach to implementing optical synapses using PCM involves constructing silicon-on-insulator (SOI) structures containing both strip and ring waveguides, with GST deposited on the ring waveguide [28]. The synaptic function is realized such that the output power is determined by the product of the transmittance at the resonant wavelength and the power of the incident light pulse. Chakraborty et al. proposed a LIF neuron architecture comprising two reversed parallel GST/add-drop silicon microrings [25], as depicted in Figure 4b. This architecture serves as the prototype of a biologically plausible spiking neuron. The “write” operation of the spiking neuron is realized by the incident optical signal, causing the temperature in the GST region to change accordingly. The “read” operation is investigated through the entire GST-microring system, with outputs from the drop and through ports of the “positive” and “negative” microrings delivered to an interferometer. This architecture simulates the membrane potential of the integrate-and-fire neuron.

Drawing inspiration from the successful emulation of biological synapses and neurons using phase-change photonic memories, Feldmann et al. experimentally realized an all-optical neuron circuit. Utilizing on-chip wavelength division multiplexing technology, they demonstrated a scalable, layered architecture composed of four artificial neurons and 60 synapses, arranged such that each neuron has 15 synapses, as illustrated in Figure 4c. The synapses consist of optical waveguides, with weights achieved by a PCM unit integrated on top of the waveguide. A multiplexer, consisting of a ring resonator, is used to couple optical pulses to the integrated waveguide and direct them to the on-chip neuron. The chip’s learning capability was tested using supervised and unsupervised machine learning algorithms by providing information from the light pulses to the network. This trained optical network was eventually able to recognize patterns [26]. As this system consists of all-optical signal processing, it has faster data processing capabilities than networks composed of electronics.

Wu et al. [27] presented a programmable waveguide mode converter based on a phase gradient metasurface of GST. This phase-change metasurface mode converter (PMMC) leverages GST’s substantial refractive index change during its phase transition to control the TE0 and TE1 spatial mode conversion of the waveguide, as shown in Figure 4d. The PMMC can precisely modulate 64 distinguishable states for the mode transition fraction. The constructed 2 × 2 PMMC array implements a multimode optical convolutional neural network with programmable cores for image processing tasks, such as edge detection and pattern recognition.

**Figure 5:**
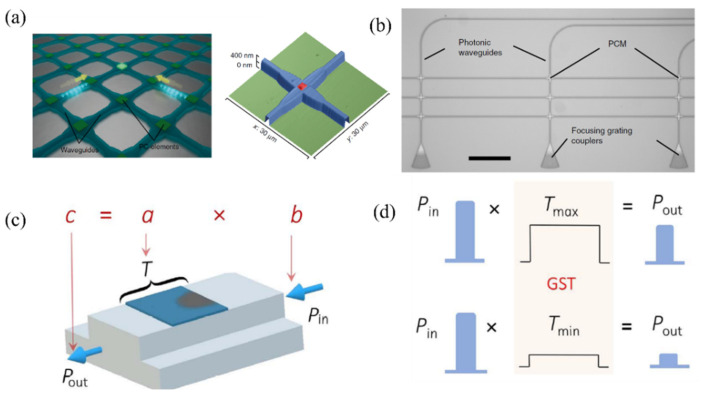
Phase change materials for in-memory computing. (**a**) Sketch of a waveguide crossing array illustrating the two-pulse addressing of individual phase-change cells. Only overlapping pulses provide sufficient power to switch the desired PCM cell [29]. (**b**) Optical micrograph of a studied crossed-waveguide photonic array [29]. (**c**) Schematic of the multiplication of two scalars (a and b), encoded in the device transmittance T and the energy of the read pulse P_in_ [30]. (**d**) The low-energy read pulse P_in_, which propagates through the device without inducing phase change, is measured at the output with an amplitude modulated by the transmittance T [30].

The concept of phase-change photonic memory for in-memory computing has also attracted significant attention. One possible scheme for performing arithmetic calculations involves designing a rectangular waveguide array, depositing PCMs at each waveguide intersection, and using the intersecting waveguides to selectively address and operate each of these basic arithmetic cells [29], as depicted in Figure 5a,b. To perform basic arithmetic calculations, the crystallinity in each PCM cell can be progressively divided into 10 different levels using a set of identical picosecond pulses. When the number of calculations reaches level 10, the PCM cell is reset to level 0 before the rest of the pulses are applied. During the resetting of the cell, a sequence of pulses is applied to a second PCM cell, which represents the next highest order multiple of the base number, similar to a progression. To perform scalar multiplication, a write pulse (P_in_) is used to program the device to a specific transmittance level (T), and then another read-out pulse (P_out_) of lower intensity, which does not change the PCM state, is implemented to detect the previous pulse-induced change in device transmittance as shown in Figure 5c,d. The multiplier “a" maps to T, and the multiplier ”b” maps to P_in_. P_out_ is the result of ”a” multiplied by ”b”. The measured values obtained from this method for choosing an arbitrary value multiplier closely match the exact value of the multiplier [30]. This result not only establishes the potential of PCM for applications in integrated photonic circuits but also validates the possibility of combining integration techniques with photonic storage and processing to enable all-photonic memory computing. In 2020, Feldmann et al. demonstrated parallelized photonic in-memory computing using phase-change material memory arrays and photonic chip-based optical frequency combs [23]. A computationally specific integrated photonic hardware accelerator (tensor core) is capable of operating at speeds of trillions of multiply-accumulate operations per second. The tensor core can be considered as the optical analogue of an application-specific integrated circuit.

## 3. The Emerging Phase Change Materials

In the case of saturable absorber-based LIF neurons, sufficient excitation causes the absorber to saturate and become transparent, resulting in the release of a pulse. This is followed by a relative refractory period of approximately 1 ns, during which the pump current restores the carrier concentration to its equilibrium value [14]. Photonic neuromorphic devices based on GST materials do have some limitations, such as high write/erasure power consumption, long response time, high system pulse energy consumption, and large link delays. These drawbacks arise from the inherent thermally induced phase-change process associated with these materials. These limitations can restrict the computational performance of neural networks. One example of a GST-based device is the non-volatile optical storage device with GST and cascaded silicon nitride microring structures. It requires an optical pulse erasure energy of 596 pJ to achieve 58% switching contrast, has a response time of 200 ns, and a reported device erase lifetime of only 50 times [13]. The GST-based all-optical pulsed neural network with four synapses and one neuron requires 525 pJ for a single learning and 200 ns for a single erase pulse width [23] This highlights the need for optimization in terms of energy consumption and response rate for future pulsed neural networks consisting of millions of neurons. Researchers have proposed an alternative material, GeTe-Sb_2_Te_3_, which undergoes a phase transition between two single crystalline states [31]. This layered phase-change material has lower structural entropy change, better erasure power consumption, improved response rate performance, and longer material lifetime compared to GST. As a result, it is more suitable for optical storage and computing functions.

**Figure 6:**
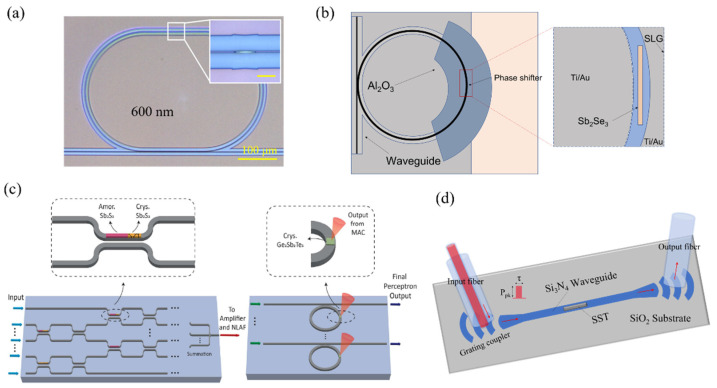
Neuromorphic devices based on variety phase change materials. (**a**) Non-volatile integrated photonic switches composed by a Ge_2_Sb_2_Se_4_Te_1_ strip on top of the SiN waveguide [32]. (**b**) Graphene-assisted phase shifter based on Sb_2_Se_3_ in a microring [35]. (**c**) All-chalcogenide optical perceptron model composed of Sb_2_S_3_ and GST [36]. (**d**) Schematic diagram of integrated photonic memory device based on Sc_0.2_Sb_2_Te_3_ [37].

As mentioned before, GST-based materials exhibit high absorption in the near-infrared band, which limits the size and scalability of GST-based neuromorphic devices. This issue makes it necessary to add additional optical amplifiers to the link, increasing power consumption and system complexity. Therefore, researchers have explored alternative phase-change materials with reduced loss in the near-infrared band to overcome these limitations. Ge_2_Sb_2_Se_4_Te_1_ (GSST) is one such material that demonstrates reduced loss in the near-infrared band, with device insertion loss less than 0.5 dB in the amorphous state [32], as illustrated in Figure 6a. Another example is the combination of GSST materials with silicon-based Mach–Zehnder interferometer structures using electrode heating, as reported by Miscuglio et al. [33]. Sb_2_S_3_/Sb_2_Se_3_ is another class of phase-change materials suitable for integrated photonics [34]. These materials have a variation ∆k of the imaginary part of the refractive index less than 10^−5^ in the 1550 nm band. Preliminary research on the hybrid integration of Sb_2_S_3_/Sb_2_Se_3_ with silicon waveguides has shown low-loss advantages, with negligible material introduction loss in the amorphous state and minimal loss in the crystalline state. In 2022, Fang et al. demonstrated a graphene-assisted non-volatile phase shifter using the low-loss PCM Sb_2_Se_3_, with a graphene monolayer as a local heater [35], as depicted in Figure 6b. An Sb_2_S_3_-tuned AlN directional coupler was also reported, showing low insertion losses and cross talk in the 770–830 nm wavelength range [36]. A three-layer deep neural network model was used to test the feasibility of the network scheme, achieving a maximum training accuracy of 94.5%. The combination of Sb_2_S_3_-programmed MZI weights with the nonlinear response of Ge_2_Sb_2_Te_5_ to femtosecond pulses was found to be sufficient for performing energy-efficient all-optical neural classifications at rates greater than 1 GHz, as depicted in Figure 6c. In 2022, Chen et al. demonstrated the use of scandium-doped antimony telluride, Sc_0.2_Sb_2_Te_3_ (SST), in neuromorphic photonic memory devices [37] as illustrated in Figure 6d. SST has been rationally designed to reduce the stochasticity of nucleation, resulting in improved crystallization speed compared to typical PCMs like GST, which have ultrafast amorphization speeds but much slower crystallization speeds. In the work by Chen et al., the optical transmission of the silicon nitride waveguide was modulated by the SST film through evanescent coupling, exhibiting low transmission for the crystalline state and high transmission for the amorphous state. By utilizing SST, they managed to significantly improve the crystallization speed, with a single 2 ns optical pulse being used in the erase process for on-chip phase-change photonic memory. The use of SST as a phase-change material in neuromorphic photonic memory devices demonstrates the potential to overcome the limitations of traditional PCMs, such as GST, by offering faster crystallization speeds. This development may lead to further improvements in photonic neuromorphic device performance and energy efficiency as research and development continue.

The phase transition process of GSST, Sb_2_S_3_/Sb_2_Se_3_, and Sc_0.2_Sb_2_Te_3_ materials still have limitations, including erase power consumption, response speed, and material lifetime, as they are based on the reversible transition between single crystal/amorphous states. Exploring the combination of phase change materials with low absorption coefficients, no melting process, and low structural entropy change with silicon-based optoelectronics is essential for solving the current problems of photonic pulse neural networks, such as power consumption, response time, and scalability.

In_2_Se_3_ material is a layered material with multiple single crystalline states, capable of undergoing reversible phase change between single crystalline states without a melting process and low structural entropy [38,39]. The material has a large optical bandgap and a low absorption coefficient in the near-infrared band [40]. In_2_Se_3_-based nonvolatile photonic devices have the potential for low erasure power consumption, fast response rate, long device lifetime, and low on-chip insertion loss. They are excellent candidates for overcoming the current bottlenecks in computational performance and scale-up of photonic pulsed neural networks. Electrical memories based on In_2_Se_3_ crystalline/amorphous transition have been reported since 2005 [41,42], and the first electro-phase transition between In_2_Se_3_ crystalline states was reported in 2017 [43]. In recent years, ferroelectric properties of α-In_2_Se_3_ thin films have been reported, which can also be used for non-volatile memory devices [44].

**Figure 7:**
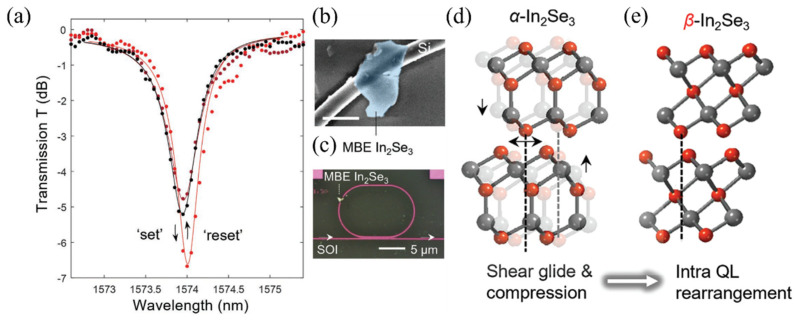
Nonvolatile all-optical memory in epitaxial In_2_Se_3_–silicon microring resonators. (**a**) Normalized transmission spectra for the hybrid resonator with *α*- (black), *β*- (red), and retrieved *α*-state (dark red) In_2_Se_3_. The dots represent experimental data, and the curves are coupled-mode-theory fittings. (**b**) Scanning electron microscope image of the as-prepared molecular beam epitaxy (MBE) film transferred onto a silicon photonic single-mode waveguide. (**c**) Optical microscopy image of an In_2_Se_3_–Si microring device. (**d**) Atomic structure of *α*-In_2_Se_3_ at room temperature (semitransparent) and after thermally activated shear-glide (solid color). The outer Se atoms fall into the interstitial sites, leading to compression of the interlayer distance. (**e**) Subsequent intra-quintuple-layer (QL) rearrangement results in the *β*-In_2_Se_3_ structure. For clarity, only the middle two QLs are included here.

In 2022, Li et al. reported a non-volatile photonic device based on In_2_Se_3_/silicon microring resonators [45], as shown in Figure 7, achieving reversible resonance shifts using normal-incidence laser pulses. On the device level, nonvolatile all-optical switching is demonstrated in molecular beam epitaxial (MBE)-grown In_2_Se_3_-silicon microring resonators with infrared light-excited photo-thermal effects. Thermal release tape was initially applied to the as-grown MBE chalcogenide film on sapphire substrates. The film could be easily peeled off with the tape. Microring devices were patterned on a SOI substrate, followed by a thin layer of poly(methyl methacrylate) (PMMA) being spin-coated onto the SOI substrate. This layer was then patterned using e-beam lithography to expose the microring and prevent residual adhesion in other areas during the transfer process. Next, the film adhered to the tape was applied to the patterned SOI substrate. The sample was baked at 110 °C for several tens of seconds, causing the thermal release tape to cure and detach from the MBE film, leaving the sample on the target substrate. Finally, an acetone bath removed the PMMA and any unwanted flakes. The covered length of the transferred α-state flake on the silicon single-mode waveguide is around 1.5 µm, as depicted in Figure 7b,c. Transition into the *β*-state was achieved at an exposure energy of 0.25 nJ. The resonance wavelength red-shifted by 100 pm, and the extinction ratio increased from 4.45 dB to 6.27 dB. With the linear coupled-mode-theory fitting of those transmission spectra, the extracted intrinsic quality factors switched between 4800 (*α*-state) and 7500 (*β*-state), and the coupling quality factor remained around 5000. When the exposure energy increased to approximately 0.56 nJ, the resonance peak shifted back, and the extinction ratio decreased to the original value. The transistor arrays defined on the thin film measured thermally excited resistivity switching from 10^6^ to 10^0^ Ω cm. Temperature-dependent in situ high-energy x-ray diffraction and derived pair distribution functions reveal that the structural phase transitions between these two layered structures are achieved through ”inter-layer shear glide” and In-Se bond rearrangement, as shown in Figure 7d,e. Differential scanning calorimetry measured reversible phase transition temperatures at around 220 °C, compared to the melting temperature beyond 600 °C in Sb-based optical phase change materials (O-PCMs). Optical transparency at telecommunication wavelengths is predicted by first-principles density functional theory calculations. The 0.81 eV fundamental bandgap difference between the two states suggests sufficient contrast in their refractive index and conductivity, according to Moss’s rule and Kramers–Kronig relations. The measured complex refractive index spectra in MBE-grown continuous thin films confirm the optical bandgaps of both states are beyond 1 eV. The polymorphic O-PCM’s optical transparency at telecommunication wavelengths and low entropic switching may overcome power and speed bottlenecks for integrated photonic in-memory computing systems.

## 4. The Comparison between Phase Change Materials

Table 1 presents a comparison of performance metrics for various O-PCMs, including Ge_2_Sb_2_Te_5_, Ge_2_Sb_2_Se_4_Te_1_, Sb_2_Se_3_, Sc_0.2_Sb_2_Te_3_, and In_2_Se_3_. The material properties are typically characterized in thin O-PCM films, and optically induced phase transitions are measured at the device level. Information from the available literature has been compiled in the table.

The most commonly used phase-change material, GST, exhibits the largest refractive index difference compared to others at 1550 nm. However, the absorption loss is relatively high for both crystalline and amorphous states, which is detrimental to large-scale integration. GSST has a significantly lower absorption loss, but a smaller refractive index difference. Although Sc_0.2_Sb_2_Te_3_ demonstrates a shorter recrystallization time compared to other antimony-based chalcogenides and chalcogenide alloys, such as Ge_2_Sb_2_Te_5_, Ge_2_Sb_2_Se_4_Te_1_, and Sb_2_Se_3_, its absorption loss remains high compared to other phase-change materials. In_2_Se_3_’s low-entropy crystalline-to-crystalline phase transitions do not involve recrystallization, resulting in a shorter phase-change time compared to conventional antimony-based chalcogenides. However, fabricating high-quality single crystalline In_2_Se_3_ is more costly than other options. Ge_2_Sb_2_Se_4_Te_1_, Sb_2_Se_3_, Sb_2_S_3_, and In_2_Se_3_ exhibit much lower loss than Ge_2_Sb_2_Te_5_ at 1550 nm, making them suitable for large-scale integration. However, the low absorption loss of these materials in the C-band prevents the optical pump signal (write/erase signal) from operating at the same wavelength as the probe signal. This limitation can impede network feedback, particularly for unsupervised machine learning applications.

To date, there is no ideal phase-change material for neuromorphic photonics. Developing new PCMs or engineering existing ones to exhibit lower absorption losses, faster switching times, and a higher contrast between refractive indices could improve device efficiency, energy consumption, and scalability. Optimizing the design of devices and systems to work efficiently with existing PCMs, such as separating the wavelengths for write/erase and read signals, may enhance network feedback, and facilitate unsupervised machine learning applications. Combining PCMs with other materials or technologies that provide complementary advantages, such as saturable absorbers or electro-optic materials, could enable the creation of more efficient and scalable devices. Developing novel fabrication methods to produce high-quality PCMs and devices with improved performance and cost-effectiveness is essential. Additionally, designing neuromorphic photonic systems that can adapt to the inherent limitations of PCMs, such as using feedback loops, error-correction mechanisms, or self-calibration techniques, may optimize performance.

## 5. Conclusions

In this work, we have provided a comprehensive review of neuromorphic photonic devices that utilize PCMs and silicon photonics technology. These devices have the potential to address the limitations of traditional spiking neural networks in terms of scalability, response delay, and energy consumption. By overcoming these bottlenecks, they may significantly enhance computing efficiency, memory capacity, input/output operations, and energy consumption management in artificial intelligence and high-performance computing fields. We have examined and compared the optical properties of various PCMs, highlighting their unique characteristics and suitability for specific applications. These comparisons have demonstrated the diverse range of possibilities that PCMs offer for designing neuromorphic photonic devices. Although there is no ideal phase-change material for neuromorphic photonics to date, several potential solutions can improve the performance of these devices. Developing new materials or engineering existing ones to exhibit lower absorption losses, faster switching times, and higher contrasts between refractive indices could significantly enhance device efficiency and scalability. Furthermore, optimizing device design to work efficiently with existing materials and exploring the integration of complementary materials or technologies may enable the creation of more efficient and scalable devices.

In conclusion, the ongoing development of neuromorphic photonic devices based on PCMs and silicon photonics technology holds great promise for revolutionizing the fields of artificial intelligence and high-performance computing. The integration of different PCMs with silicon-based optoelectronics could pave the way for more energy-efficient and high-performance photonic neuromorphic devices. Nevertheless, further research and development are essential to optimize these materials, address their limitations, and unlock their full potential. As the field progresses, we anticipate that innovative solutions will emerge, leading to the realization of advanced neuromorphic photonic systems that can transform computing paradigms and reshape the landscape of artificial intelligence applications.

## Figures and Tables

**Table 1:** Comparison of optical phase change materials and correspondent device performance.

Parameters	Ge_2_Sb_2_Te_5_	Ge_2_Sb_2_Se_4_Te_1_	Sb_2_Se_3_	Sc_0.2_Sb_2_Te_3_	In_2_Se_3_
$\left|∆n\right|$@1550 nm	3.34 [33]	2.0 [33]	0.77 [34]	~0.68 (20 nm) [37] ~0.21 (10 nm)	0.45 [45]
k@1550 nm	k_C_ = 1.882 [33] k_A_ = 0.192 [33]	k_C_ = 0.350 [33] k_A_ = 1.8 × 10^−4^ [33]	k_C_ = ~0 [34] k_A_ = ~0 [34]	k_C_ = ~1.23 (20 nm), ~0.94 (10 nm) [37] k_A_ = ~0.99 (20 nm), ~0.83 (10 nm) [37]	k*_α_* = 0 [45] k*_β_* = 0 [45]
Optical bandgap (eV)	~0.7 [46]	0.3 [32]	1.6–2.1 [34]	Not mentioned	*α*: 1.44 [45] *β*: 1.27 [45]
Phase change temperature (^o^C)	T_Melting_ = ~620 [47] T_crystallization_ = 200 [48]	T_Melting_ > 627 [33] T_Crystallization_~250 [33]/400 [32]	T_Melting_ = 611 [49] T_Crystallization_ ~200 [34]	T_Crystallization_~170 [50]	T*_α_*_→*β*_~200 [45] T*_β_*_→*β*‘_~200 [51] T*_β_*_‘→*α*_~60 [51]
Exposed laser energy or energy density	A→C: ~1 nJ [52]	C→A: 13.6 nJ [32] A→C: 408 nJ [32]	C→A: ~5 nJ/μm^2^ [34] A→C: ~200 μJ/μm^2^ [34]	Not mentioned	*α*→*β*: ~5 nJ/μm^2^ [45] *β*→*α*: ~11 nJ/μm^2^ [45]
Average power (mW)	A→C: >10 [52]	C→A: 136 [32] A→C: 136 [32]	C→A: > 55 [34] A→C: > 45 [34]	Not mentioned	*α*→*β*: 4 [45] *β*→*α*: 8.7 [45]
Spot size/wavelength	Unknown spot size/λ = 658 nm [52]	Unknown spot size/λ = 633 nm + 780 nm [32]	C→A: 1.5 μm [34], A→C: 1.8 μm [34]/λ = 638 nm	Not mentioned	10 μm/λ = 1064 nm [45]
Exposure time (ns)	C→A: < 1 [53] A→C: 10^2^ [52]	C→A: 10^2^ [32] A→C: 3 × 10^6^ [32]	C→A: > 2 × 10^2^ [34] A→C: 10^7^ [34]	C→A: 2 [36] A→C: 2 [36]	*α*→*β*: 15 [45] *β*→*α*: 15 [45]

C: Crystalline; A: Amorphous.

## Data Availability

In this review manuscript, no new data were created.
